# Exploring the Relationship between Persons with Hearing Loss/Deafness and Their Hearing Dogs

**DOI:** 10.3390/ani14111527

**Published:** 2024-05-22

**Authors:** Carlie J. Driscoll, Jessica Hill, Anna Torre, Nancy Pachana

**Affiliations:** 1 Animal-Assisted Interventions Research Alliance, University of Queensland, Brisbane, QLD 4072, Australia; jessica.hill@uq.edu.au (J.H.); a.torre@uq.net.au (A.T.); 2 School of Psychology, University of Queensland, Brisbane, QLD 4072, Australia; n.pachana@psy.uq.edu.au

**Keywords:** animal-assisted services, assistance dogs, attachment, deafness, hearing dogs, hearing loss

## Abstract

**Simple Summary:**

Hearing Dogs are assistance animals who provide practical help to their owners with hearing loss/Deafness by alerting them to environmental sounds. While some studies have verified the contribution of Hearing Dogs to their owners’ quality of life, little is known about the relationship that is established between canine and owner. This survey study investigated the anticipated role of Hearing Dogs in 23 prospective owners, as well as emotional attachment in 58 Hearing Dog owners. Results revealed that prospective owners expected the dog to play a broad supportive role in their lives, beyond simple assistance with sounds. For owners, a strong emotional component was evident in their relationships with the Hearing Dogs, particularly in the “people substitution” category. This study suggests that the relationship expectations of potential Hearing Dog recipients should be achievable, as evidenced by the strong relationships reported by owners.

**Abstract:**

The reported positive outcomes of animal-assisted services have led to an emerging interest in many different aspects of human–animal interactions. The influence of an assistance animal is thought to encompass several psychosocial domains in the life of a person with a significant health impairment. However, little is known about the mechanisms underlying the relationship between Hearing Dogs and their owners. A prospective study design using a written questionnaire method was utilized to survey 58 current and 23 prospective Australian Lions Hearing Dogs owners. The Pet Expectations Inventory (PEI) was used to investigate the anticipated role of Hearing Dogs in waitlisted persons with hearing loss/Deafness, whereas the Lexington Attachment to Pets Scale (LAPS) was completed by current owners to assess emotional attachment. The results revealed a high mean PEI score (M = 73.1, SD = 10.9, Mdn = 73.0, range: 55–91), with prospective owners strongly expecting the role of Hearing Dogs to include companionship/love and security. Furthermore, strong attachment features were evident in the owners’ relationships with Hearing Dogs, as demonstrated by a high total LAPS score (M = 81.2, SD = 7.5, range: 63–91). Mean scores for statements within the “people substitution” category were highest (range = 3.6/4.00–3.9/4.00). In this demographically homogenous study cohort, it appeared that the high expectations of potential Hearing Dog owners for their animals to serve supportive roles beyond hearing assistance should be achievable, as evidenced by the strong attachment relationships displayed between Hearing Dogs and their owners.

## 1. Introduction

Hearing loss has been recognized as an important health issue by the World Health Organization [1] due to the impact on a person’s physical and social functioning, as well as emotional wellbeing. Globally, approximately 5% or 466 million people have disabling hearing loss, with this number expected to increase to over 900 million people by 2050 [1]. Hearing loss can impact a person’s communication and, subsequently, their employment, education, and ability to engage socially. Research has consistently found that people with hearing loss are more likely to experience social isolation, low self-esteem, and depression [2]. Increased awareness of the effects of hearing loss has seen growing interest in interventions that consider the widespread psychosocial needs of individuals. 

Current research in animal-assisted interventions (i.e., animal-assisted therapy, animal-assisted learning/education, and animal-assisted activities) suggests functional, socio-emotional, and health benefits can be gained from interactions with animals [3,4,5]. In the related but distinct field of animal-assisted services, the work of assistance animals has also been shown to provide their owners (synonymous with handlers) with similar gains. These animals are specifically trained to support alleviation of their owner’s disability. For persons with hearing loss/Deafness, the provision of a Hearing Dog may be appropriate. Hearing Dogs are professionally trained to alert owners to important environmental sounds such as a smoke alarm, doorbell ring, oven timer, et cetera. Growing research on Hearing Dogs suggests the psychosocial benefits gained from ownership may preface increases in an individual’s overall wellbeing and health-related quality of life [6]. However, the nature of and mechanisms behind the relationship between owner and Hearing Dog have not been fully explored. 

Central to the owners’ interaction with the Hearing Dogs is the human–animal relationship. The connection or bond experienced between owner and dog is thought to act as a resource of social support and comfort that positively influences health [7]. Although no single or unified theory has been identified to describe the psychosocial effects that result from interaction between humans and dogs, human-human attachment theory has been suggested to partially explain the affective-emotional features of the human-animal relationship [8]. Attachment encompasses behaviours that relate to feelings of a safe haven, a secure base, and proximity seeking to an attachment figure [9]. 

It has long been thought that dogs can serve as attachment figures for humans, with the human–dog relationship sharing several features of the human–human relationship [10]. For instance, the protection and security offered by dogs to their owners is akin to the safe haven and secure base behaviours seen in human-;human attachment [10,11]. For Hearing Dogs specifically, development of the secure relationship may be partly explained by the protection and security abilities of the dog, as they alert the owners to everyday sounds [12,13].

Several researchers have begun to explore the relationship between assistance dog handlers and their dogs through an attachment lens. White, Mills, and Hall [14] found that people who had insecure attachments in other relationships benefitted more from the relationship with an assistance dog. Those with an anxious attachment reported a higher quality of life than those with an avoidant attachment style. Furthermore, within an interview study, Kwong and Bartholomew [12] aimed to examine the attachment attributes of assistance dog handlers who had established and, subsequently, lost a relationship with an assistance dog. Findings from this study found that although the attachment dynamics (safe haven, secure base, separation anxiety) were viewed by participants as important factors influencing the closeness they felt to their dog, the benefits received from ‘reciprocal caregiving’ were perceived as equally important. Although participants valued the safety and security they felt in the presence of their assistance dog, their ability to care for their dog by tending to their needs was perceived to be equally as valuable in supporting their relationship. 

The limited literature concerning the relationships between assistance dogs and their owners suggests that several complex processes are at play. Further research into the nature of the Hearing Dog-owner relationship could provide helpful insight into the needs of persons with hearing loss/Deafness, assist in the formation of realistic expectations, and offer implications for relationship development. This study aimed to characterise the relationships formed between persons with hearing loss/Deafness and their Hearing Dogs by considering expectations prior to ownership and experiences following ownership. It was hypothesised that the nature and function of the relationship would have strongly emotional and supportive aspects, extending beyond the practical aspects of Hearing Dog ownership to include typical human-to-human attachment features.

## 2. Materials and Methods

### 2.1. Sample

The study was conducted with the assistance of the Australian Lions Hearing Dogs program. The Australian Lions Hearing Dogs is the only accredited provider in Australia that trains and places Hearing Dogs. The selection criteria for the current study comprises the eligibility for Hearing Dog placement listed below. Provider placement requirements include evidence of a severe or profound degree of hearing loss and need for a hearing assistance dog. Medical reports are required to ensure an individual is physically able to care for the dog, and a character reference and statement of financial capacity ensure the safety and comfort of the dog. All current (*n* = 105) and waitlisted (*n* = 45) clients were invited to participate. Participation was voluntary and responses were anonymised. The current study builds upon two previous projects involving the same participant groups as reported in Drewitt-Smith et al. [6] and Singh, Driscoll, and Pachana [15]. 

A total of 58 current owners (15 males, 43 females), ranging in age from 32 to 89 years (M = 65.1 years; SD = 11.7), agreed to participate in the study, providing a response rate of 55%. Initially, 24 waitlisted individuals opted in, and 1 participant later chose to withdraw. A total of 23 waitlisted individuals (5 males, 18 females), ranging in age from 25 to 88 years (M = 62.6 years; SD = 14.7), participated in the study, resulting in a final response rate of 51%.

### 2.2. Design and Procedure

A prospective study design using a written survey method was chosen for this study. This type of design permitted all current and waitlisted clients of the Australian Lion Hearing Dogs to be included in the study in an efficient and cost-effective manner. Recruitment was accomplished with the help of the Australian Lion Hearing Dogs using a postal survey approach. A survey pack was sent via post or email; all individuals were also given the option to complete the surveys by phone (using an assistive listening service—TTY). Participants who wished to use the postal option were given pre-paid, self-addressed return envelopes. In addition, participants received a reminder through social media and a newsletter three weeks following the distribution of the surveys. 

Group comparison design through an examination of survey data was utilised to compare data obtained from the questionnaires of prospective Hearing Dog owners with those who had previously received a Hearing Dog.

### 2.3. Instruments

Each participant was posted a survey pack that included six questionnaires, a participant information and consent form, and a return envelope. The full package of questionnaires can be viewed in the Supplementary Materials, File S1. It was expected that the completion of the package would require less than 1 h of the participant’s time. The questionnaires of relevance to the current study included:

*The General Information Survey* is predominantly a closed-ended demographic survey given to owners and waitlisted participants. The questionnaire was created explicitly for this study, containing questions relating to the participant’s age, gender, marital status, education, living environment, employment status, current pets, and previous Hearing Dog or pet ownership. 

*The Hearing Information Survey* is primarily a closed-ended questionnaire designed for this study to explore the audiological characteristics of all participants. It contained questions regarding the: type, degree, and duration of the hearing loss; preferred communication mode; participation in the Deaf community; types of supports used; and amplification device details. A section was added for participants in the waitlisted group that included questions regarding the types of sounds individuals needed help with. Similarly, a section with closed-ended questions titled “about your Hearing Dog” was added for the owner cohort to explore the types of sound the Hearing Dog assisted with, and an option to list other sounds owners required help with. All participants were also asked to provide a copy of their audiogram.

*The Pet Expectations Inventory (PEI)* is a 13-question instrument given to potential owners on the current Australian Lions waitlist to investigate the roles a Hearing Dog is expected to play in the lives of prospective owners. The PEI measure was adapted from the Kidd, Kidd, and George’s [16] questionnaire developed for pet adoptions. Each participant was required to give responses on a 7-point Likert scale on how much they agreed or disagreed with each statement. Scores ranged from 1 (“strongly disagree”) to 7 (“strongly agree”). A total score can be obtained between 13 and 91 points. Higher scores indicate the responder expects the Hearing Dog to serve a broad role in addition to the required practical assistance.

*Lexington Attachment to Pets Scale (LAPS)* is a 23-item psychometric questionnaire of an individual’s emotional attachment to a pet and addresses known variables that contribute to the human–animal bond. This questionnaire was completed by the owner cohort only. Like all pet-related surveys, it does not provide direct information on human attachment styles, but provides insight into attachment features of the relationship between human and animal. The statements can be allocated into three subscales: “general attachment” (Items 1–6), “animal rights” (Items 7–14), and “people substitution” (Items 15–23). The questionnaire was chosen for the current research, as it has good internal consistency and has been validated by its authors (Cronbach’s alpha 0.928), who initially employed it to assess pet attachment orientation [17]. Responses were coded, as in Johnson et al. [17], using a 4-point Likert Scale ranging from 1 (“Strongly disagree”) to 4 (“Strongly agree”), with scores reversed for statements 8 and 21. Total scores can range from 23 to 92, with higher scores indicating a higher emotional attachment.

### 2.4. Analysis of Data

The data obtained from the questionnaires were analysed in IBM SPSS Statistics version 26. Statistical analyses were performed using descriptive statistics (e.g., mean, median, SD), and distributions between the groups were assessed using Chi-square analysis. The significance level was set at *a* = 0.05. 

## 3. Results

Completed questionnaires were received for 58 individuals in the owner cohort and 23 waitlisted individuals. The demographic characteristics of participants are listed in Table 1. Participants were predominantly female (75.3%), retired (67.9%), and lived in a home that they owned (65.9%). Roughly half of all participants lived alone (54.9%). The mean age of participants in the owner group was 65.1 years (SD = 11.7, Mdn = 67 years, range: 32–89 years). The mean age of participants in the waitlist group was 62.6 years (SD = 14.7, Mdn = 66 years, range: 25–88 years). Most participants did not currently own a pet (64.2%), but had owned a pet in the past (91.4%). The vast majority of participants had not previously owned a Hearing Dog (74.1%). Chi-squared analysis revealed a significant difference in education level between the groups (X^2^ = 5.40, df = 1, *p* = 0.020), with a higher proportion of participants in the waitlist cohort completing TAFE/college and university education. There was also a significant difference in homeownership distributions between the two cohorts. The owner cohort had a higher rate of reported homeownership (X^2^ = 18.67, df = 1, *p* = 0.00001) compared to the waitlisted cohort. The mean duration of Hearing Dog ownership was 5.2 years (SD = 4.3), and most owners reported that the Hearing Dog attends them everywhere they go (76%).

The audiological characteristics of the waitlisted and owner participants are shown in Table 2. Participants’ hearing loss was predominantly sensorineural (53.1%). A large proportion of participants did not know the type of their hearing loss (23.5%). The degree of hearing loss was mostly profound (51.9%), followed by severe (42.0%). Most participants had acquired a hearing loss gradually over time (63.0%). A large proportion of participants communicated orally only (75.3%), and a few participants were members of the Deaf community (19.8%). The most frequent form of support used in the past was hearing aids (95.1%) followed by assisted listening devices (60.5%), with 42.0% of participants having used cochlear implants. Over half of the participants reported that they currently used hearing aids (65.4%) for more than 12 h a day (61.0%), with 50.6% of participants presently using assistive listening devices. The distributions between the waitlisted and owner cohort were similar except for the variables of past support group attendance and cochlear implant use (X^2^ = 4.52, df = 1, p = 0.033; and X^2^ = 5.40, df = 1, *p* = 0.02). A larger proportion of owner participants had previously attended a support group and used a cochlear implant. Chi-square analysis showed that a significantly larger proportion of owner participants reported their hearing aids/cochlear implants usefulness as very useful (X^2^ = 8.97, df = 1, *p* = 0.002) and extremely useful (X^2^ = 5.60, df = 1, *p* = 0.012). No other significant differences were found in the remaining audiological characteristics (*p* > 0.05) between groups. Most frequently (>80%), the waitlist cohort expected to receive Hearing Dog assistance with detecting mobile phones, door knocks, bells, and smoke alarms. The owner group reported that assistance was most commonly provided for the same sounds, with the exception that the dog was more helpful in detection of oven sounds than mobile phones. 

### 3.1. Pet Expectations Inventory (PEI)

The PEI scores of the waitlisted cohort are summarised in Table 3. Scores indicated how strongly waitlisted participants agreed with 13 statements on a 7-point scale. Distributions for all items were generally skewed towards higher scores (median scores of 6 or 7 for 10/13 items) indicating strong agreement with statements. The lowest score was obtained for the item “I expect to teach my dog tricks” (median score = 4). The mean score for the total PEI questionnaire was 73.1 out of a maximum total score of 91 (SD = 10.9, Mdn = 73.0, range: 55–91).

### 3.2. Lexington Attachment to Pets Scale (LAPS)

Table 4 outlines the LAPS scores of the owner cohort. The total mean LAPS score was 81.2 (SD = 7.5, range: 63–91), which sits within the upper range of possible scores and is indicative of strong emotional attachment. Owner response distributions for means in all three categories were clearly skewed towards higher scores (≥3, apart from the mean for the reverse score statement #8), indicating agreement/strong agreement. For the reverse score statements (#8 and #21), the expected effect of lower scores was not evident. Mean scores for statements within the “people substitution” category were highest (range = 3.6–3.9), followed by those in the “animal rights” category (range = 3.2–3.7), followed by the “general attachment category” (range = 3.0–3.6). 

## 4. Discussion

To our knowledge, this was the first study to explore the attachment relationship between Hearing Dogs and their owners. We found that the expectations of prospective owners for the Hearing Dog to serve a broad socio-emotional role in their lives was brought to fruition by ownership, with owners displaying a very strong attachment relationship with their dogs. 

### 4.1. Demographic Characteristics

Overall, demographic details of the participants suggest that they likely possessed the financial stability and time required to care for a Hearing Dog, and understood the implications of ownership, including the responsibilities required in the daily care of an animal. There were significant differences in the demographic variables between owner and waitlist groups for education level and homeownership only. Therefore, it was concluded that the groups were largely homogenous in nature. 

### 4.2. Audiological Characteristics

Of note, most participants had a profound sensorineural hearing loss acquired gradually over time and had tried hearing aids (95%); however, only half reported current use of hearing aids, which suggests that they either had no need or positive preference for the devices or that they had remaining needs that could not be addressed by amplification. The waitlist cohort expected the Hearing Dog to aid in detecting a wide range of environmental sounds, and it was noted that the most common of these expected sounds were generally the same ones that owners reported as being successfully identified by the dog. Our study also revealed significant differences between the cohorts in satisfaction with hearing aids or cochlear implants. Given the overall homogeneity of the total participant cohort, it is possible that this increased perception of hearing aid benefit in the owners could be attributed to the indirect effects in socio-emotional domains provided by Hearing Dog ownership, rather than improved satisfaction with hearing per se. Further research could specifically examine perceptions of hearing and aided benefit pre- and post-Hearing Dog placement.

### 4.3. Expected Roles of Hearing Dogs

The PEI scores obtained from the waitlisted group revealed that participants expected the Hearing Dog to serve an expansive role in their lives, beyond assistance in alerting to environmental sounds. Participants rated very highly the expectation statements that related to receiving companionship, giving and receiving love/affection, and the provision of security. Although minimal literature has addressed the expectations of potential Hearing Dog recipients, Hart and colleagues’ [18] study of 23 prospective owners found that the perceived companionship/love offered by a Hearing Dog was the most common reason for pursuing ownership (78.3%), followed by assistance with hearing (56.5%), and then enhanced security (43.5%). The findings of the latter and current study suggest that prospective owners expect the dog to fill the role of an attachment figure, sharing similar aspects as seen in human-human social relationships. Understandably, such could appeal to persons with hearing loss/Deafness due to their higher risk of reduced social networks, loneliness, difficulties with verbal communication, social distress, and decreased independence [19].

### 4.4. Relationship between Hearing Dogs and Owners

The LAPS questionnaire’s total scores for current Hearing Dog owners showed that a very strong emotional attachment was present, with the “people substitution” domain producing the highest ratings. Thus, it appears that Hearing Dogs can serve as an alternative or adjunct to human-human social relationships, through the companionship that they provide, as also observed by Kurdek [10] for pet dogs. Hart et al. [18] also found love/companionship to be the primary benefit reported by 86.8% of their 38 Hearing Dog owners. Companionship has been described in the literature as the primary human benefit gained from living with an animal [20]. For pet owners, the literature has shown that those who score highly on pet attachment measures are more likely to have reduced social networks and a more extensive history of negative life events [21,22]. The same could feasibly be the case for many persons with severe or profound hearing loss/Deafness due to communication limitations, social restrictions, and loneliness which increase with degree of loss [2]. In addition, older pet owners with strong attachment to their pets have been shown to have less human social support [21,22]. Indeed, Hearing Dog owners in the current study were aged 65 years on average, and persons with hearing loss are at a higher risk of experiencing loneliness than those with normal hearing, implying reduced social support.

Some authors have posed that it is not the ownership aspect of an animal that yields improvements in wellbeing, but rather the degree of attachment to the animal [23]. Attachment has been described as a significant factor in human–animal relationships and as a major contributor to the positive benefits gained from interactions with animals [5,8,10,24]. Furthermore, recent research has begun to explore factors suggested to positively influence the development of this attachment between owner and dog [13,25]. Within their survey study, Hill and colleagues [25] reported that participants who perceived themselves to spend the most time with their dog engaging in shared meaningful occupations also reported a stronger human–dog attachment. Within the present study, most participants reported spending a significant amount of their day with their hearing dog, potentially supporting the development of their bond. 

### 4.5. Limitations

The limitations of a survey approach include an inability to account for individual interpretations or misunderstanding of questions. Such may have been an issue within the current study, as it appeared that participants did not pay close attention to the completion of LAPS survey questions presented in reverse-score format. In addition, the survey pack provided to all participants mainly consisted of self-report items measuring participants’ subjective reports, which may not be an accurate measure of relationship features. Furthermore, this was a unidirectional investigation that did not consider the dogs’ relational or attachment behaviours. Additionally, there exists a possibility of participant bias, as those with a strong relationship with their Hearing Dog may have been more likely to engage with the study. It is also known that females are more likely than males to report high attachment to their companion animals, and the current investigation’s cohort were predominantly women.

Various validated questionnaires have been used in the literature to define the human–animal relationship in animal-assisted interventions. However, inherent difficulties arise in defining the socio-emotional aspects of a relationship with animals. It requires individuals to use terms and concepts commonly applied to human developmental psychology and to assume that the meaning remains the same when transferred to the human–animal bond. Lewis [26] argued that terms within behavioural ecology would be more appropriate when describing the human–canine bond, as there is no compelling evidence that this bond is synonymous, either neuropsychologically or socially, with an infant–caregiver attachment. 

Finally, the response rate of 55% obtained in the present study signifies sufficient representation of Hearing Dog recipients in Australia. Nonetheless, reducing the total number of surveys to be completed at once [27], along with offering an electronic version of the surveys, may have improved the participation rate. 

## 5. Conclusions

This study attempted to quantify the attachment aspects of the Hearing Dog-owner relationship by using validated questionnaires to measure expectations of ownership and attachment strength. Potential owners strongly expected the Hearing Dog to offer companionship, love/affection, and security. Existing owners rated their attachment to their Hearing Dogs very strongly, particularly in the domain of people substitution. Thus, expectations of ownership, largely characterized by companionship, appear to be appropriately validated by the strong attachment relationships displayed between Hearing Dogs and their owners. 

## Figures and Tables

**Table 1:** Sociodemographic characteristics of the owner cohort (*n* = 58) and the waitlist cohort (*n* = 23).

	Waitlist		Owner		Total	
	N	%	N	%	N	%
**Gender**						
Female	18	78.3	43	74.1	61	75.3
Male	5	21.7	15	25.9	20	24.7
**Age**						
<65 years	10	43.4	26	44.8	36	44.4
≥65 years	12	52.2	29	50	41	50.6
Missing Data	1	4.3	3	5.2	4	4.9
**Marital Status**						
Single	3	14.3	15	25.9	18	22.2
Partnered	1	4.8	2	3.4	3	3.7
Married	6	28.6	17	29.3	23	28.4
Separated	1	4.8	2	3.4	3	3.7
Divorced	4	19	15	25.9	19	23.5
Widowed	6	28.6	7	12.1	13	16
Missing data	2	8.7	0	0	2	2.5
**Education Completed**						
Primary School	2	9.1	4	7	6	7.4
High School	5	22.7	29	50.9	34	42
College/TAFE	8	36.4	14	24.6	22	27.2
University	7	31.8	10	17.5	17	20.7
Missing Data	1	4.3	1	1.7	2	2.4
**Living Arrangement**						
Alone	13	59.1	32	56.1	45	54.9
Someone else	9	40.9	25	43.9	34	41.5
Missing Data	1	4.3	1	1.7	2	2.4
**Accommodation**						
House	20	87	41	71.9	61	74.4
Unit	3	13	16	28.1	19	23.2
Missing Data	0	0	1	1.7	1	1.2
**Homeowner**						
Yes	18	78.3	36	67.9	54	65.9
No	5	21.7	17	32.1	22	26.8
Missing Data	0	0	5	8.6	5	6.1
**Employment Status**						
Retired	16	69.6	39	73.6	55	67.9
Employed	7	30.4	14	26.4	21	25.9
Missing Data	0	0	5	8.6	5	6.2
**Previous Pet Ownership**						
Yes	21	91.3	53	91.4	74	91.4
No	2	8.7	5	8.6	7	8.6
**Previous Hearing Dog Ownership**						
Yes	2	8.7	18	31	20	24.7
No	20	87	40	69	60	74.1
Missing Data	1	1.2	0	0	1	1.2
**Current Pet Ownership**						
Yes	9	40	14	26.4	23	28.4
No	13	59.1	39	73.6	52	64.2
Missing Data	1	4.3	5	8.6	6	7.4

**Table 2:** Audiological characteristics of the owner cohort (*n* = 58) and waitlist cohort (*n* = 23).

	Waitlist		Owner		Total	
	N	%	N	%	N	%
**Type of Hearing loss**						
Sensorineural	9	39.1	34	58.6	43	53.1
Conductive	2	8.7	0	0	2	2.5
Mixed	4	17.4	9	15.5	13	16
Do not know/unsure	6	26.1	13	22.4	19	23.5
Missing Data	2	8.7	2	3.4	4	4.9
**Degree of Hearing Loss**						
Severe	11	47.8	23	39.7	34	42
Profound	10	43.5	32	55.2	42	51.9
Do not know/Unsure	2	8.7	3	5.2	5	6.2
**Duration of Hearing Loss**						
Present since birth	7	30.4	20	34.5	27	33.3
Gradual over time	16	69.6	35	60.3	51	63
Sudden due to illness/trauma	0	0	3	5.2	3	3.7
**Communication Mode**						
Oral (speech) only	18	78.3	43	74.1	61	75.3
Sign Language only	0	0	5	8.6	5	6.2
Oral and Sign Language	5	21.7	10	17.2	15	18.5
**Member of Deaf Community**						
Yes	5	21.7	11	19	16	19.8
No	18	78.3	44	75.9	62	76.5
Missing Data	0	0	3	5.2	3	3.7
**Previous Supports Used**						
Hearing aids	23	100	54	93.1	77	95.1
Cochlear Implants	5	21.7	29	50	34	42
Hearing loss support group	0	0	10	17.2	10	12.3
Communication training	4	17.4	22	37.9	26	23.2
Assistive listening devices	13	56.5	36	62.1	49	60.5
Other	0	0	8	13.8	8	9.9
**Current Supports Used**						
Hearing aids	17	73.9	36	62.1	53	65.4
Cochlear implants	7	30.4	27	46.6	34	42
Hearing loss support group	0	0	2	3.4	2	2.5
Communication training	2	8.7	7	12.1	9	11.1
Assistive listening devices	11	47.8	30	51.7	41	50.6
Other	1	4.3	11	19	12	14.4
**Daily Use of Devices**						
7–12 h	9	39.1	17	29.3	26	31.7
>12 h	14	60.9	36	62	50	61
Missing Data	0	0	5	8.6	5	6.2
**Usefulness of Devices**						
Not useful at all	0	0	2	3.4	2	2.5
A little bit useful	4	17.4	4	6.9	8	9.9
Useful	7	30.4	11	19	18	22.2
Very Useful	5	21.7	34	58.6	39	48.1
Extremely Useful	6	26.1	4	6.9	10	12.3
Missing Data	1	4.3	3	5.2	4	4.9
**Sounds Required/Sounds Assisted**						
Clock	12	52.2	35	60.3	47	58
Oven	11	47.8	50	86.2	61	75.3
Baby	3	13	7	12.1	10	12.3
Mobile	19	82.6	35	60.3	54	66.7
Phone	15	65.2	27	46.6	42	51.9
Door	22	95.7	56	96.6	78	96.3
Bell	21	91.3	48	82.8	69	85
Kettle	5	21.7	7	12.1	12	14.8
Smoke alarm	21	91.3	55	94.8	7	93.8
Get person	15	65.2	25	43.1	40	49.4

**Table 3:** Pet Expectations Inventory scores for the waitlist cohort (*n* = 22) *. Statements scored on a seven-point Likert scale (strongly disagree to strongly agree) are arranged from highest to lowest mean score.

Statement	Mean (SD)	Median
I expect my dog to be a living thing for me to love.	6.6 (0.9)	7
I expect the dog to be a companion for me.	6.6 (0.8)	7
I expect to talk to my dog.	6.4 (0.9)	7
I expect my dog to love me.	6.1 (1.2)	7
I expect to stroke and cuddle my dog.	6.1 (1.1)	7
I expect the dog always to be there for me.	6.1 (1.0)	6
I expect my dog to make me feel better when I am sad or discouraged.	5.8 (1.2)	6
I expect my dog to protect me.	5.7 (1.5)	6
I expect my dog to be an interesting topic of conversation with friends and relatives.	5.6 (1.5)	6
I expect to play with my dog.	5.4 (1.8)	6
I expect my dog to be a source of laughter.	5.2 (1.7)	5
I expect to confide in my dog.	4.6 (2.4)	5
I expect to teach my dog tricks.	4.0 (2.0)	4
**Total Score**	**73.1 (10.9)**	**73**

* Missing data from one participant.

**Table 4:** Lexington Attachment to Pets Scale scores for the owner cohort (*n* = 58). Statements are scored on a four-point Likert scale (strongly disagree to strongly agree).

	Statement	Mean (SD)
**General attachment**		
1	My dog means more to me than any of my friends.	3.1 (0.8)
2	Quite often I confide in my dog.	3.0 (0.9)
3	I believe that dogs should have the same rights and privileges as family members.	3.2 (0.8)
4	I believe my dog is my best friend.	3.6 (0.6)
5	Quite often, my feelings toward people are affected by the way they react to my dog.	3.3 (0.7)
6	I love my dog because he/she is more loyal to me than most of the people in my life.	3.1 (0.8)
**Animal Rights**		
7	I enjoy showing other people pictures of my dog.	3.2 (0.9)
8	I think my dog is just a dog.	3.0 (1.1)
9	I love my dog because it never judges me.	3.4 (0.8)
10	My dog knows when I am feeling bad.	3.6 (0.6)
11	I often talk to other people about my dog.	3.6 (0.6)
12	My dog understands me.	3.5 (0.5)
13	I believe that loving my dog helps me stay healthy.	3.6 (0.6)
14	Dogs deserve as much respect as humans do.	3.7 (0.4)
**People Substitution**		
15	My dog and I have a very close relationship.	3.9 (0.3)
16	I would do almost anything to take care of my dog.	3.8 (0.5)
17	I play with my dog quite often.	3.6 (0.6)
18	I consider my dog to be a great companion.	3.9 (0.3)
19	My dog makes me feel happy.	3.8 (0.4)
20	I feel that my dog is a part of my family.	3.9 (0.4)
21	I am not very attached to my dog.	3.9 (0.7)
22	Owning a dog adds to my happiness.	3.8 (0.5)
23	I consider my dog to be a friend.	3.8 (0.4)
	**Mean Total score**	**81.2 (7.5)**

## Data Availability

De-identified data are available from the corresponding author, upon request.

## Supplementary Materials

The following supporting information can be downloaded at: https://www.mdpi.com/article/10.3390/ani14111527/s1, File S1: Full Questionnaire Package.
