# Supplementary material for: Exploring the Relationship between Persons with Hearing Loss/Deafness and Their Hearing Dogs
Source: Animals (Basel). 2024 May 22;14(11):1527. doi: 10.3390/ani14111527 (PMC11171399; doi:10.3390/ani14111527)
Supplement: Supplementary file 1 [file animals-14-01527-s001.zip › animals-2981906-supplementary.pdf]

THE UNIVERSITY OF QUEENSLAND
AUSTRALIA

School of Health and Rehabilitation Sciences
AUDIOLOGY DIVISION

Head of Division:
Assoc/Prof. Wayne Wilson

The University of Queensland
Brisbane Qld 4072 Australia
Telephone (07) 3365 1797
International +61 7 3365 1797
Facsimile +61 7 3365 1788
Internet www.shrs.uq.edu.au

PROJECT TITLE: Hearing Dogs – Effects on Hearing Handicap, Relationships, & Quality of Life

PARTICIPANT INFORMATION SHEET

As an owner of an Australian Lions Hearing Dog, you are invited to take part in the above named research project. Please be advised that you are not obliged in any way to participate should you not wish to. Your participation is completely voluntary and will not affect your relationship with the University of Queensland or Australian Lions.

Aim: This project aims to investigate the effects of owning a Hearing Dog on hearing handicap, relationships, and Quality-of-Life, in a large Australian cohort.

We hope that this project will improve understanding of the real benefits of these service animals, and contribute to improvements in service provision. The project will also raise community and professional awareness of the benefits of Hearing Dogs for persons with hearing loss.

Your involvement in this study: By consenting to participate in this study, you are agreeing to complete the following questionnaires via reply-paid post. There are no other activities for you to complete other than these surveys.

- General Information Survey
- Hearing Information Survey
- Hearing Handicap Inventory
- Social Functioning Questionnaire
- Medical Outcome Survey
- Lexington Attachment to Pets Scale or Pet Expectations Inventory

We would be grateful if you could return your completed surveys in the reply-paid post envelope within 5 weeks' time.
If you would prefer to complete these via email or phone (instead of post), please contact Associate Professor Carlie Driscoll (Ph. 0401587640 Email: carlie.driscoll@uq.edu.au).

Confidentiality and storage of data: Your data will be treated by the researchers in the strictest confidence and used only for the purposes of the study. Your anonymity will be respected - you will not be identified in any resulting reports or publications. All information will be stored on a password protected computer file accessible only to the researchers or in locked filing cabinets. All data will be kept for seven years from the completion of the project before being destroyed.

Feedback available to you: The findings of this study will be submitted for publication in the academic literature and presented at related conferences. You are welcome to contact the research team if you would like to obtain a copy of these papers.

Participation & Withdrawal: Your participation is voluntary and you are free to withdraw at any time. To withdraw from the project, please contact Mr David Horne (CEO of Australian Lions Hearing Dogs -

Ph. 08 8388 7836 Email: ceo@lionshearingdogs.com.au) as soon as possible. Participation in, or withdrawal from, this research project will have no effect on your hearing health care or any other service. Nor will participation be of any direct benefit to you. You will not receive any reimbursement or other enticements for participation. There are no risks associated with your participation in this project, other than the inconvenience associated with completing the surveys.

Ethical Clearance: This study adheres to the Guidelines of the ethical review process of The University of Queensland and the National Statement on Ethical Conduct in Human Research. Whilst you are free to discuss your participation in this study with project staff (Assoc/Prof Carlie Driscoll Ph. 0401587640), if you would like to speak to an officer of the University not involved in the study, you may contact the Ethics Coordinators on +617 3365 3924 / +617 3443 1656 or email humanethics@research.uq.edu.au

Thank you for considering participation in this research.

Chief Investigators from The University of Queensland:
Associate Professor Carlie Driscoll Ph. 0401587640 Email: carlie.driscoll@uq.edu.au
Professor Nancy Pachana Ph. 07 3365 6832 Email: n.pachana@psy.uq.edu.au

PLEASE RETAIN THIS INFORMATION SHEET FOR YOUR OWN RECORDS

THE UNIVERSITY OF QUEENSLAND
AUSTRALIA

School of Health and Rehabilitation Sciences
AUDIOLOGY DIVISION

Head of Division:
Assoc/Prof. Wayne Wilson

The University of Queensland
Brisbane Qld 4072 Australia
Telephone (07) 3365 1797
International +61 7 3365 1797
Facsimile +61 7 3365 1788
Internet www.shrs.uq.edu.au

PROJECT TITLE: Hearing Dogs – Effects on Hearing Handicap, Relationships, & Quality of Life

PARTICIPANT CONSENT FORM

I ....
[PLEASE PRINT NAME USING BLOCK CAPITALS]
hereby consent to participate in the above named research study.

1. I have read the information provided.
2. The purpose and benefits of the project have been explained to me.
3. I understand that there may be no direct benefits to me from participating.
4. Details of procedures and any risks have been explained to my satisfaction.
5. I have had the opportunity to discuss taking part in this research.
6. I understand that the information gained in this project may be published as explained.
7. I understand that my confidentiality will be maintained and that I will not be identified and all data will be securely stored during and after the project.
8. I understand that I am free to withdraw consent to further involvement in the research without consequence at any time.

Name: ____

Signature: ____

Date: ____________________

Project Team:
Associate Professor Carlie Driscoll Ph. 0401587640 Email: carlie.driscoll@uq.edu.au
Professor Nancy Pachana Ph. 07 3365 6832 Email: n.pachana@psy.uq.edu.au

IF YOU WISH TO PARTICIPATE IN THIS STUDY, PLEASE FOLLOW THESE STEPS:

1. **SIGN THIS CONSENT FORM**
2. **PUT THE CONSENT FORM INTO THE PLAIN ENVELOPE MARKED 'CONSENT FORM'**
3. **SEAL THE PLAIN ENVELOPE**
4. **PLACE THE PLAIN ENVELOPE INTO THE LARGE, REPLY-PAID ENVELOPE**
5. **PLACE THE COMPLETED SURVEYS INTO THE LARGE REPLY-PAID ENVELOPE**
6. **PUT THE REPLY-PAID ENVELOPE INTO ANY AUSTRALIA POST BOX.**

THE UNIVERSITY OF QUEENSLAND
AUSTRALIA

GENERAL INFORMATION SURVEY

ABOUT ME	
**Age:**	___________years (please provide your current age)
**Gender:**	☐ Male ☐ Female ☐ Prefer not to say
**Marital status:**	☐ Single ☐ Partnered ☐ Married ☐ Separated ☐ Divorced ☐ Widowed
**Education Completed:**	☐ Primary School ☐ High School ☐ College/TAFE ☐ University
**Living with:**	☐ Partner ☐ Children ☐ Partner and children ☐ Other family ☐ Friends ☐ Flatmate ☐ Alone
**Living in:**	☐ House ☐ Townhouse ☐ Unit/Apartment
**Home Ownership:**	☐ Owned ☐ Mortgaged/Home Loan ☐ Government Housing
**Employment:**	☐ Full-time ☐ Part-time ☐ Casual ☐ Unemployed ☐ Retired
**Have you owned a pet before (not a Hearing Dog)?**	☐ Yes ☐ No
**Current pets:**	☐ Cat ☐ Bird ☐ Reptile ☐ Other ☐ None
**ABOUT MY HEARING DOG**	
**Dog Age:**	_________________years (please provide approximate age)
**Dog Breed:**	___________________ (please provide, if known)
**For how long have you owned this Hearing Dog?**	_______years _______mths (please give approximate time of ownership)
**Is he/she your first Hearing Dog?**	☐ Yes ☐ No
**Where does your Hearing Dog go?**	☐ Everywhere that I go ☐ Some places that I go ☐ Stays at home only
**EXPERIENCES WITH YOUR HEARING DOG**	
Please tell us about your experiences with a Hearing Dog – the good, the bad, and anything that needs changing	
**Positive things about having a Hearing Dog:**	
**Negative things about having a Hearing Dog:**	
**Changes that you recommend:**	

THE UNIVERSITY OF QUEENSLAND AUSTRALIA

HEARING INFORMATION SURVEY

ABOUT YOUR HEARING LOSS	
**Type of Hearing Loss:**	☐ Sensorineural (inner ear/nerves) ☐ Conductive (outer/middle ear) ☐ Mixed<br>☐ Don't know/unsure
**Degree of Hearing Loss:**	☐ Severe ☐ Profound ☐ Don't know/unsure
**Time with Hearing Loss:**	☐ Hearing loss present since birth<br>☐ Gradual hearing loss over time<br>☐ Sudden hearing loss due to illness/trauma
**Communication Mode:**	☐ Oral (speech) only ☐ Sign language only ☐ Mixture of speech and signing
**Are you a member of the Deaf community?**	☐ Yes ☐ No
**Have you ever used any of the following supports in the past?**	☐ Hearing Aids ☐ Cochlear Implant ☐ Hearing Loss support group<br>☐ Communication training (for lip-reading, listening strategies, etc.)<br>☐ Assistive Listening Devices (e.g., Headphones, vibrating alarm, flashing light alarm)<br>☐ Other: ________________ (please specify)
**Do you currently use any of the following supports?**	☐ Hearing Aids ☐ Cochlear Implant ☐ Hearing Loss support group<br>☐ Communication training (for lip-reading, listening strategies, etc.)<br>☐ Assistive Listening Devices (e.g., Headphones, vibrating alarm, flashing light alarm)<br>☐ Other: ________________ (please specify)
**How long do you wear your hearing aids/cochlear implant for each day?**	☐ 1-2 hours<br>☐ 3-6 hours<br>☐ 7-12 hours<br>☐ More than 12 hours<br>☐ I don't wear any hearing devices
**How useful are your hearing aids/cochlear implant?**	☐ Not useful at all<br>☐ A little bit useful<br>☐ Useful<br>☐ Very useful<br>☐ Extremely useful<br>☐ I don't wear any hearing devices. Please tell us why you do not wear any:<br>________________<br>________________<br>________________
**ABOUT YOUR HEARING DOG**	
**Which sounds does your Hearing Dog help you with?**	☐ Alarm clock<br>☐ Oven timer<br>☐ Baby cry<br>☐ Mobile phone<br>☐ House phone<br>☐ Door knock<br>☐ Door bell<br>☐ Kettle whistle<br>☐ Smoke alarm<br>☐ Getting another person<br>☐ Other: ________________ (please list the sounds)

PLEASE TURN OVER THE PAGE

**Are there any other sounds that you would like help with?**	☐ Yes:______________________________ (please list the sounds)<br>______________________________<br>______________________________<br>☐ No
**MY AUDIOGRAM**	
If you have a copy of your hearing test results (audiogram), please attach it to this survey.	

THE UNIVERSITY OF QUEENSLAND
AUSTRALIA

HEARING HANDICAP INVENTORY FOR THE ELDERLY

THIS SURVEY IS FOR PERSONS AGED 65 YEARS AND OLDER.
IF YOU ARE UNDER 65 YEARS OF AGE, PLEASE SKIP THIS SURVEY AND MOVE TO THE NEXT QUESTIONNAIRE.

S1	Does a hearing problem cause you to use the phone less often than you would like?	☐ Yes ☐ No ☐ Sometimes
E2	Does a hearing problem cause you to feel embarrassed when meeting new people?	☐ Yes ☐ No ☐ Sometimes
S3	Does a hearing problem cause you to avoid groups of people?	☐ Yes ☐ No ☐ Sometimes
E4	Does a hearing problem make you irritable?	☐ Yes ☐ No ☐ Sometimes
E5	Does a hearing problem cause you to feel frustrated when talking to members of your family?	☐ Yes ☐ No ☐ Sometimes
S6	Does a hearing problem cause you difficulty when attending a party?	☐ Yes ☐ No ☐ Sometimes
E7	Does a hearing problem cause you to feel "stupid" or "dumb"?	☐ Yes ☐ No ☐ Sometimes
S8	Do you have difficulty hearing when someone speaks in a whisper?	☐ Yes ☐ No ☐ Sometimes
E9	Do you feel handicapped by a hearing problem?	☐ Yes ☐ No ☐ Sometimes

S10	Does a hearing problem cause you difficulty when visiting friends, relatives, or neighbours?	☐ Yes ☐ No ☐ Sometimes
S11	Does a hearing problem cause you to attend religious services less often than you would like?	☐ Yes ☐ No ☐ Sometimes
E12	Does a hearing problem cause you to be nervous?	☐ Yes ☐ No ☐ Sometimes
S13	Does a hearing problem cause you to visit friends, relatives, or neighbours less often than you would like?	☐ Yes ☐ No ☐ Sometimes
E14	Does a hearing problem cause you to have arguments with family members?	☐ Yes ☐ No ☐ Sometimes
S15	Does a hearing problem cause you difficulty when listening to TV or radio?	☐ Yes ☐ No ☐ Sometimes
S16	Does a hearing problem cause you to go shopping less often than you would like?	☐ Yes ☐ No ☐ Sometimes
E17	Does any problem or difficulty with your hearing upset you at all?	☐ Yes ☐ No ☐ Sometimes
E18	Does a hearing problem cause you to want to be by yourself?	☐ Yes ☐ No ☐ Sometimes
S19	Does a hearing problem cause you to talk to family members less often than you would like?	☐ Yes ☐ No ☐ Sometimes
E20	Do you feel that any difficulty with your hearing limits or hampers your personal or social life?	☐ Yes ☐ No ☐ Sometimes
S21	Does a hearing problem cause you difficulty when in a restaurant with relatives or friends?	☐ Yes ☐ No ☐ Sometimes

E22	Does a hearing problem cause you to feel depressed?	☐ Yes ☐ No ☐ Sometimes
S23	Does a hearing problem cause you to listen to TV or radio less often than you would like?	☐ Yes ☐ No ☐ Sometimes
E24	Does a hearing problem cause you to feel uncomfortable when talking to friends?	☐ Yes ☐ No ☐ Sometimes
E25	Does a hearing problem cause you to feel left out when you are with a group of people?	☐ Yes ☐ No ☐ Sometimes

Ventry, I. M., & Weinstein, B. E. (1982). The Hearing Handicap Inventory for the Elderly: A new tool. *Ear Hear, 3*, 128-134.

THE UNIVERSITY OF QUEENSLAND
AUSTRALIA

HEARING HANDICAP INVENTORY FOR ADULTS

THIS SURVEY IS FOR PERSONS AGED UNDER 65 YEARS.
IF YOU ARE OLDER THAN 65 YEARS OF AGE, PLEASE SKIP THIS SURVEY AND MOVE TO THE NEXT QUESTIONNAIRE.

S1	Does a hearing problem cause you to use the phone less often than you would like?	☐ Yes ☐ No ☐ Sometimes
E2	Does a hearing problem cause you to feel embarrassed when meeting new people?	☐ Yes ☐ No ☐ Sometimes
S3	Does a hearing problem cause you to avoid groups of people?	☐ Yes ☐ No ☐ Sometimes
E4	Does a hearing problem make you irritable?	☐ Yes ☐ No ☐ Sometimes
E5	Does a hearing problem cause you to feel frustrated when talking to members of your family?	☐ Yes ☐ No ☐ Sometimes
S6	Does a hearing problem cause you difficulty when attending a party?	☐ Yes ☐ No ☐ Sometimes
E7	Does a hearing problem cause you to feel frustrated when talking to coworkers, clients, or customers?	☐ Yes ☐ No ☐ Sometimes
S8	Do a hearing problem cause you difficulties in the movies or theater?	☐ Yes ☐ No ☐ Sometimes
E9	Do you feel handicapped by a hearing problem?	☐ Yes ☐ No ☐ Sometimes

S10	Does a hearing problem cause you difficulty when visiting friends, relatives, or neighbours?	☐ Yes ☐ No ☐ Sometimes
S11	Does a hearing problem cause you difficulty hearing/understanding coworkers, clients, or customers?	☐ Yes ☐ No ☐ Sometimes
E12	Does a hearing problem cause you to be nervous?	☐ Yes ☐ No ☐ Sometimes
S13	Does a hearing problem cause you to visit friends, relatives, or neighbours less often than you would like?	☐ Yes ☐ No ☐ Sometimes
E14	Does a hearing problem cause you to have arguments with family members?	☐ Yes ☐ No ☐ Sometimes
S15	Does a hearing problem cause you difficulty when listening to TV or radio?	☐ Yes ☐ No ☐ Sometimes
S16	Does a hearing problem cause you to go shopping less often than you would like?	☐ Yes ☐ No ☐ Sometimes
E17	Does any problem or difficulty with your hearing upset you at all?	☐ Yes ☐ No ☐ Sometimes
E18	Does a hearing problem cause you to want to be by yourself?	☐ Yes ☐ No ☐ Sometimes
S19	Does a hearing problem cause you to talk to family members less often than you would like?	☐ Yes ☐ No ☐ Sometimes
E20	Do you feel that any difficulty with your hearing limits or hampers your personal or social life?	☐ Yes ☐ No ☐ Sometimes
S21	Does a hearing problem cause you difficulty when in a restaurant with relatives or friends?	☐ Yes ☐ No ☐ Sometimes

E22	Does a hearing problem cause you to feel depressed?	☐ Yes ☐ No ☐ Sometimes
S23	Does a hearing problem cause you to listen to TV or radio less often than you would like?	☐ Yes ☐ No ☐ Sometimes
E24	Does a hearing problem cause you to feel uncomfortable when talking to friends?	☐ Yes ☐ No ☐ Sometimes
E25	Does a hearing problem cause you to feel left out when you are with a group of people?	☐ Yes ☐ No ☐ Sometimes

Ventry, I. M., & Weinstein, B. E. (1982). The Hearing Handicap Inventory for the Elderly: A new tool. *Ear Hear, 3*, 128-134.

THE UNIVERSITY OF QUEENSLAND
AUSTRALIA

SOCIAL FUNCTION QUESTIONNAIRE

PLEASE LOOK AT THE STATEMENTS BELOW AND TICK THE REPLY THAT COMES CLOSEST TO HOW YOU HAVE BEEN RECENTLY

I complete my tasks at work and home satisfactorily.	☐ Most of the time<br>☐ Quite often<br>☐ Sometimes<br>☐ Not at all	0<br>1<br>2<br>3
I find my tasks at work and at home very stressful.	☐ Most of the time<br>☐ Quite often<br>☐ Sometimes<br>☐ Not at all	3<br>2<br>1<br>0
I have no money problems.	☐ No problems at all<br>☐ Slight worries only<br>☐ Definite problems<br>☐ Very severe problems	0<br>1<br>2<br>3
I have difficulties in getting and keeping close relationships.	☐ Severe difficulties<br>☐ Some problems<br>☐ Occasional problems<br>☐ No problems at all	3<br>2<br>1<br>0
I get on well with my family and other relatives.	☐ Yes, definitely<br>☐ Yes, usually<br>☐ No, some problems<br>☐ No, severe problems	0<br>1<br>2<br>3
I feel lonely and isolated from other people.	☐ Almost all the time<br>☐ Much of the time<br>☐ Not usually<br>☐ Not at all	3<br>2<br>1<br>0
I enjoy my spare time.	☐ Very much<br>☐ Sometimes<br>☐ Not often<br>☐ Not at all	0<br>1<br>2<br>3

Tyrer, P., Nur, U., Crawford, M., Karlsen, S., Mclean, C., Rao, B., & Johnson, T. (2005). The social functioning questionnaire: A rapid and robust measure of perceived functioning. *International Journal of Social Psychiatry*, *51*(3), 265-275.

THE UNIVERSITY OF QUEENSLAND
AUSTRALIA

MEDICAL OUTCOME SURVEY (MOS SF-36)

For each of the following questions, please circle the number that best describes your answer.

**1. In general, would you say your health is:**	
Excellent	1
Very good	2
Good	3
Fair	4
Poor	5
**2. Compared to one year ago,**	
Much better now than one year ago	1
Somewhat better now than one year ago	2
About the same	3
Somewhat worse now than one year ago	4
Much worse now than one year ago	5

3. The following items are about activities you might do during a typical day. **Does your health now limit you** in these activities? If so, how much? (Circle One Number on Each Line)

	**Yes, Limited a lot (1)**	**Yes, Limited a little (2)**	**No, Not limited at all (3)**
a. **Vigorous activities**, such as running, lifting heavy objects, participating in strenuous sports	1	2	3
b. **Moderate activities**, such as moving a table, pushing a vacuum cleaner, bowling, or playing golf	1	2	3
c. Lifting or carrying groceries	1	2	3
d. Climbing **several** flights of stairs	1	2	3
e. Climbing **one** flight of stairs	1	2	3
f. Bending, kneeling, or stooping	1	2	3

g. Walking **more than a mile**	1	2	3
h. Walking **several blocks**	1	2	3
i. Walking **one block**	1	2	3
j. Bathing or dressing yourself	1	2	3

4. During the **past 4 weeks**, have you had any of the following problems with your work or other regular daily activities **as a result of your physical health**?
(Circle One Number on Each Line)

	**Yes (1)**	**No (2)**
a. Cut down the amount of time you spent on work or other activities	1	2
b. **Accomplished less** than you would like	1	2
c. Were limited in the **kind** of work or other activities	1	2
d. Had **difficulty** performing the work or other activities (for example, it took extra effort)	1	2

5. During the **past 4 weeks,** have you had any of the following problems with your work or other regular daily activities **as a result of any emotional problems** (such as feeling depressed or anxious)? (Circle One Number on Each Line)

	**Yes**	**No**
a. Cut down the amount of time you spent on work or other activities	1	2
b. **Accomplished less** than you would like	1	2
c. Didn't do work or other activities as **carefully** as usual	1	2

**6. During the past 4 weeks, to what extent has your physical health or emotional problems interfered with your normal social activities with family, friends, neighbors, or groups?**	
Not at all	1
Slightly	2
Moderately	3
Quite a bit	4
Extremely	5

7. How much bodily pain have you had during the past 4 weeks?	
None	1
Very mild	2
Mild	3
Moderate	4
Severe	5
Very severe	6
**8. During the past 4 weeks, how much did pain interfere with your normal work (including both work outside the home and housework)?**	
Not at all	1
A little bit	2
Moderately	3
Quite a bit	4
Extremely	5

These questions are about how you feel and how things have been with you **during the past 4 weeks**. For each question, please give the one answer that comes closest to the way you have been feeling. (Circle One Number on Each Line)

9. How much of the time during the **past 4 weeks** . . .

	All of the time	Most of the time	A good bit of the time	Some of the time	A little of the time	None of the time
a. Did you feel full of pep?	1	2	3	4	5	6
b. Have you been a very nervous person?	1	2	3	4	5	6
c. Have you felt so down in the dumps that nothing could cheer you up?	1	2	3	4	5	6
d. Have you felt calm and peaceful?	1	2	3	4	5	6
e. Did you have a lot of energy?	1	2	3	4	5	6

	All of the time	Most of the time	A good bit of the time	Some of the time	A little of the time	None of the time
f. Have you felt downhearted and blue?	1	2	3	4	5	6
g. Did you feel worn out?	1	2	3	4	5	6
h. Have you been a happy person?	1	2	3	4	5	6
i. Did you feel tired?	1	2	3	4	5	6

**10. During the past 4 weeks, how much of the time has your physical health or emotional problems interfered with your social activities (like visiting with friends, relatives, etc.)? (Circle One Number)**	
All of the time	1
Most of the time	2
Some of the time	3
A little of the time	4
None of the time	5

11. How **TRUE or FALSE** is each of the following statements for you. (Circle One Number on Each Line)

	Definitely True	Mostly True	Don't Know	Mostly False	Definitely False
a. I seem to get sick a little easier than other people	1	2	3	4	5
b. I am as healthy as anybody I know	1	2	3	4	5
c. I expect my health to get worse	1	2	3	4	5
d. My health is excellent	1	2	3	4	5

Ware JE, Snow KK, Kosinski M, Gandek B. *SF-36® Health Survey Manual and Interpretation Guide*. Boston, MA: New England Medical Center, The Health Institute, 1993.

THE UNIVERSITY OF QUEENSLAND
AUSTRALIA

LEXINGTON ATTACHMENT TO PETS SCALE

FOR EACH STATEMENT, CHECK WHETHER YOU STRONGLY AGREE, SOMEWHAT AGREE, SOMEWHAT DISAGREE, OR STRONGLY DISAGREE.

		STRONGLY AGREE	SOMEWHAT AGREE	SOMEWHAT DISAGREE	STRONGLY DISAGREE
A.	My dog means more to me than any of my friends.				
B.	Quite often I confide in my dog.				
C.	I believe that dogs should have the same rights and privileges as family members.				
D.	I believe my dog is my best friend.				
E.	Quite often, my feelings toward people are affected by the way they react to my dog.				
F.	I love my dog because he/she is more loyal to me than most of the people in my life.				
G.	I enjoy showing other people pictures of my dog.				
H.	I think my dog is just a dog.				
I.	I love my dog because it never judges me.				
J.	My dog knows when I'm feeling bad.				
K.	I often talk to other people about my dog.				
L.	My dog understands me.				
M.	I believe that loving my dog helps me stay healthy.				
N.	Dogs deserve as much respect as humans do.				
O.	My dog and I have a very close relationship.				
P.	I would do almost anything to take care of my dog.				
Q.	I play with my dog quite often.				
R.	I consider my dog to be a great companion.				
S.	My dog makes me feel happy.				
T.	I feel that my dog is a part of my family.				
U.	I am not very attached to my dog.				
V.	Owning a dog adds to my happiness.				
W.	I consider my dog to be a friend.				

Johnson, T.P Garrity, T.F. & Stallones, L. (1992). Psychometric Evaluation of the Lexington Attachment to Pets Scale (Laps), *Anthrozoös*, 5:3, 160-175.

THE UNIVERSITY OF QUEENSLAND
AUSTRALIA

School of Health and Rehabilitation Sciences
AUDIOLOGY DIVISION

Head of Division:
Assoc/Prof. Wayne Wilson

The University of Queensland
Brisbane Qld 4072 Australia
Telephone (07) 3365 1797
International +61 7 3365 1797
Facsimile +61 7 3365 1788
Internet www.shrs.uq.edu.au

PROJECT TITLE: Hearing Dogs – Effects on Hearing Handicap, Relationships, & Quality of Life

PARTICIPANT INFORMATION SHEET

As a potential owner of an Australian Lions Hearing Dog, you are invited to take part in the above named research project. Please be advised that you are not obliged in any way to participate should you not wish to. Your participation is completely voluntary and will not affect your relationship with the University of Queensland or Australian Lions.

Aim: This project aims to investigate the effects of owning a Hearing Dog on hearing handicap, relationships, and Quality-of-Life, in a large Australian cohort.

We hope that this project will improve understanding of the real benefits of these service animals, and contribute to improvements in service provision. The project will also raise community and professional awareness of the benefits of Hearing Dogs for persons with hearing loss.

Your involvement in this study: By consenting to participate in this study, you are agreeing to complete the following questionnaires via reply-paid post. There are no other activities for you to complete other than these surveys.

- General Information Survey
- Hearing Information Survey
- Hearing Handicap Inventory
- Social Functioning Questionnaire
- Medical Outcome Survey
- Lexington Attachment to Pets Scale or Pet Expectations Inventory

We would be grateful if you could return your completed surveys in the reply-paid post envelope within 5 weeks' time.
If you would prefer to complete these via email or phone (instead of post), please contact Associate Professor Carlie Driscoll (Ph. 0401587640 Email: carlie.driscoll@uq.edu.au).

Confidentiality and storage of data: Your data will be treated by the researchers in the strictest confidence and used only for the purposes of the study. Your anonymity will be respected - you will not be identified in any resulting reports or publications. All information will be stored on a password protected computer file accessible only to the researchers or in locked filing cabinets. All data will be kept for seven years from the completion of the project before being destroyed.

Feedback available to you: The findings of this study will be submitted for publication in the academic literature and presented at related conferences. You are welcome to contact the research team if you would like to obtain a copy of these papers.

Participation & Withdrawal: Your participation is voluntary and you are free to withdraw at any time. To withdraw from the project, please contact Mr David Horne (CEO of Australian Lions Hearing Dogs -

Ph. 08 8388 7836   Email: ceo@lionshearingdogs.com.au) as soon as possible. Participation in, or withdrawal from, this research project will have no effect on your hearing health care or any other service. Nor will participation be of any direct benefit to you. You will not receive any reimbursement or other enticements for participation. There are no risks associated with your participation in this project, other than the inconvenience associated with completing the surveys.

Ethical Clearance: This study adheres to the Guidelines of the ethical review process of The University of Queensland and the National Statement on Ethical Conduct in Human Research. Whilst you are free to discuss your participation in this study with project staff (Assoc/Prof Carlie Driscoll Ph. 0401587640), if you would like to speak to an officer of the University not involved in the study, you may contact the Ethics Coordinators on +617 3365 3924 / +617 3443 1656 or email humanethics@research.uq.edu.au

Thank you for considering participation in this research.

Chief Investigators from The University of Queensland:
Associate Professor Carlie Driscoll   Ph. 0401587640   Email: carlie.driscoll@uq.edu.au
Professor Nancy Pachana   Ph. 07 3365 6832   Email: n.pachana@psy.uq.edu.au

PLEASE RETAIN THIS INFORMATION SHEET FOR YOUR OWN RECORDS

THE UNIVERSITY OF QUEENSLAND
AUSTRALIA

School of Health and Rehabilitation Sciences
AUDIOLOGY DIVISION

Head of Division:
Assoc/Prof. Wayne Wilson

The University of Queensland
Brisbane Qld 4072 Australia
Telephone (07) 3365 1797
International +61 7 3365 1797
Facsimile +61 7 3365 1788
Internet www.shrs.uq.edu.au

PROJECT TITLE: Hearing Dogs – Effects on Hearing Handicap, Relationships, & Quality of Life

PARTICIPANT CONSENT FORM

I ....
[PLEASE PRINT NAME USING BLOCK CAPITALS]
hereby consent to participate in the above named research study.

1. I have read the information provided.
2. The purpose and benefits of the project have been explained to me.
3. I understand that there may be no direct benefits to me from participating.
4. Details of procedures and any risks have been explained to my satisfaction.
5. I have had the opportunity to discuss taking part in this research.
6. I understand that the information gained in this project may be published as explained.
7. I understand that my confidentiality will be maintained and that I will not be identified and all data will be securely stored during and after the project.
8. I understand that I am free to withdraw consent to further involvement in the research without consequence at any time.

Name: ____

Signature: ____

Date: __________________

Project Team:
Associate Professor Carlie Driscoll Ph. 0401587640 Email: carlie.driscoll@uq.edu.au
Professor Nancy Pachana Ph. 07 3365 6832 Email: n.pachana@psy.uq.edu.au

IF YOU WISH TO PARTICIPATE IN THIS STUDY, PLEASE FOLLOW THESE STEPS:

1. **SIGN THIS CONSENT FORM**
2. **PUT THE CONSENT FORM INTO THE PLAIN ENVELOPE MARKED 'CONSENT FORM'**
3. **SEAL THE PLAIN ENVELOPE**
4. **PLACE THE PLAIN ENVELOPE INTO THE LARGE, REPLY-PAID ENVELOPE**
5. **PLACE THE COMPLETED SURVEYS INTO THE LARGE REPLY-PAID ENVELOPE**
6. **PUT THE REPLY-PAID ENVELOPE INTO ANY AUSTRALIA POST BOX.**

THE UNIVERSITY OF QUEENSLAND
AUSTRALIA

GENERAL INFORMATION SURVEY

ABOUT ME	
**Age:**	____________years (please provide your current age)
**Gender:**	☐ Male ☐ Female ☐ Prefer not to say
**Marital status:**	☐ Single ☐ Partnered ☐ Married ☐ Separated ☐ Divorced ☐ Widowed
**Education Completed:**	☐ Primary School ☐ High School ☐ College/TAFE ☐ University
**Living with:**	☐ Partner ☐ Children ☐ Partner and children ☐ Other family ☐ Friends ☐ Flatmate ☐ Alone
**Living in:**	☐ House ☐ Townhouse ☐ Unit/Apartment
**Home Ownership:**	☐ Owned ☐ Mortgaged/Home Loan ☐ Government Housing
**Employment:**	☐ Full-time ☐ Part-time ☐ Casual ☐ Unemployed ☐ Retired
**Have you owned a pet before (not a Hearing Dog)?**	☐ Yes ☐ No
**Current pets:**	☐ Cat ☐ Bird ☐ Reptile ☐ Other ☐ None
**Have you previously owned a Hearing Dog?**	☐ Yes ☐ No (If No, you may stop here. Do not complete the next section.)

EXPERIENCES WITH YOUR HEARING DOG	
Please tell us about your past experiences with a Hearing Dog – the good, the bad, and anything that needs changing	
**Positive things about having a Hearing Dog:**	
**Negative things about having a Hearing Dog:**	
**Changes that you recommend:**	

THE UNIVERSITY OF QUEENSLAND
AUSTRALIA

HEARING INFORMATION SURVEY

ABOUT YOUR HEARING LOSS	
**Type of Hearing Loss*:**	☐ Sensorineural (inner ear/nerves) ☐ Conductive (outer/middle ear) ☐ Mixed<br>☐ Don't know/unsure
**Degree of Hearing Loss:**	☐ Severe ☐ Profound ☐ Don't know/unsure
**Time with Hearing Loss:**	☐ Hearing loss present since birth<br>☐ Gradual hearing loss over time<br>☐ Sudden hearing loss due to illness/trauma
**Communication Mode:**	☐ Oral (speech) only ☐ Sign language only ☐ Mixture of speech and signing
**Are you a member of the Deaf community?**	☐ Yes ☐ No
**Have you ever used any of the following supports in the past?**	☐ Hearing Aids ☐ Cochlear Implant ☐ Hearing Loss support group<br>☐ Communication training (for lip-reading, listening strategies, etc.)<br>☐ Assistive Listening Devices (e.g., Headphones, vibrating alarm, flashing light alarm)<br>☐ Other: ______________________ (please specify)
**Do you currently use any of the following supports?**	☐ Hearing Aids ☐ Cochlear Implant ☐ Hearing Loss support group<br>☐ Communication training (for lip-reading, listening strategies, etc.)<br>☐ Assistive Listening Devices (e.g., Headphones, vibrating alarm, flashing light alarm)<br>☐ Other: ______________________ (please specify)
**How long do you wear your hearing aids/cochlear implant for each day?**	☐ 1-2 hours<br>☐ 3-6 hours<br>☐ 7-12 hours<br>☐ More than 12 hours<br>☐ I don't wear any hearing devices
**How useful are your hearing aids/cochlear implant?**	☐ Not useful at all<br>☐ A little bit useful<br>☐ Useful<br>☐ Very useful<br>☐ Extremely useful<br>☐ I don't wear any hearing devices. Please tell us why you do not wear any:<br>______________________<br>______________________<br>______________________
**ABOUT YOUR HEARING DOG**	
**Which sounds do you want the Hearing Dog to help you with?**	☐ Alarm clock<br>☐ Oven timer<br>☐ Baby cry<br>☐ Mobile phone<br>☐ House phone<br>☐ Door knock<br>☐ Door bell<br>☐ Kettle whistle<br>☐ Smoke alarm<br>☐ Getting another person<br>☐ Other: ______________________ (please list the sounds)

*** If you have a copy of your hearing test results (audiogram), please attach it to this survey.**

THE UNIVERSITY OF QUEENSLAND
AUSTRALIA

HEARING HANDICAP INVENTORY FOR THE ELDERLY

THIS SURVEY IS FOR PERSONS AGED 65 YEARS AND OLDER.
IF YOU ARE UNDER 65 YEARS OF AGE, PLEASE SKIP THIS SURVEY AND MOVE TO THE NEXT QUESTIONNAIRE.

S1	Does a hearing problem cause you to use the phone less often than you would like?	☐ Yes ☐ No ☐ Sometimes
E2	Does a hearing problem cause you to feel embarrassed when meeting new people?	☐ Yes ☐ No ☐ Sometimes
S3	Does a hearing problem cause you to avoid groups of people?	☐ Yes ☐ No ☐ Sometimes
E4	Does a hearing problem make you irritable?	☐ Yes ☐ No ☐ Sometimes
E5	Does a hearing problem cause you to feel frustrated when talking to members of your family?	☐ Yes ☐ No ☐ Sometimes
S6	Does a hearing problem cause you difficulty when attending a party?	☐ Yes ☐ No ☐ Sometimes
E7	Does a hearing problem cause you to feel "stupid" or "dumb"?	☐ Yes ☐ No ☐ Sometimes
S8	Do you have difficulty hearing when someone speaks in a whisper?	☐ Yes ☐ No ☐ Sometimes
E9	Do you feel handicapped by a hearing problem?	☐ Yes ☐ No ☐ Sometimes

S10	Does a hearing problem cause you difficulty when visiting friends, relatives, or neighbours?	☐ Yes ☐ No ☐ Sometimes
S11	Does a hearing problem cause you to attend religious services less often than you would like?	☐ Yes ☐ No ☐ Sometimes
E12	Does a hearing problem cause you to be nervous?	☐ Yes ☐ No ☐ Sometimes
S13	Does a hearing problem cause you to visit friends, relatives, or neighbours less often than you would like?	☐ Yes ☐ No ☐ Sometimes
E14	Does a hearing problem cause you to have arguments with family members?	☐ Yes ☐ No ☐ Sometimes
S15	Does a hearing problem cause you difficulty when listening to TV or radio?	☐ Yes ☐ No ☐ Sometimes
S16	Does a hearing problem cause you to go shopping less often than you would like?	☐ Yes ☐ No ☐ Sometimes
E17	Does any problem or difficulty with your hearing upset you at all?	☐ Yes ☐ No ☐ Sometimes
E18	Does a hearing problem cause you to want to be by yourself?	☐ Yes ☐ No ☐ Sometimes
S19	Does a hearing problem cause you to talk to family members less often than you would like?	☐ Yes ☐ No ☐ Sometimes
E20	Do you feel that any difficulty with your hearing limits or hampers your personal or social life?	☐ Yes ☐ No ☐ Sometimes
S21	Does a hearing problem cause you difficulty when in a restaurant with relatives or friends?	☐ Yes ☐ No ☐ Sometimes

E22	Does a hearing problem cause you to feel depressed?	☐ Yes ☐ No ☐ Sometimes
S23	Does a hearing problem cause you to listen to TV or radio less often than you would like?	☐ Yes ☐ No ☐ Sometimes
E24	Does a hearing problem cause you to feel uncomfortable when talking to friends?	☐ Yes ☐ No ☐ Sometimes
E25	Does a hearing problem cause you to feel left out when you are with a group of people?	☐ Yes ☐ No ☐ Sometimes

Ventry, I. M., & Weinstein, B. E. (1982). The Hearing Handicap Inventory for the Elderly: A new tool. *Ear Hear, 3*, 128-134.

THE UNIVERSITY OF QUEENSLAND
AUSTRALIA

HEARING HANDICAP INVENTORY FOR ADULTS

THIS SURVEY IS FOR PERSONS AGED UNDER 65 YEARS.
IF YOU ARE OLDER THAN 65 YEARS OF AGE, PLEASE SKIP THIS SURVEY AND MOVE TO THE NEXT QUESTIONNAIRE.

S1	Does a hearing problem cause you to use the phone less often than you would like?	☐ Yes ☐ No ☐ Sometimes
E2	Does a hearing problem cause you to feel embarrassed when meeting new people?	☐ Yes ☐ No ☐ Sometimes
S3	Does a hearing problem cause you to avoid groups of people?	☐ Yes ☐ No ☐ Sometimes
E4	Does a hearing problem make you irritable?	☐ Yes ☐ No ☐ Sometimes
E5	Does a hearing problem cause you to feel frustrated when talking to members of your family?	☐ Yes ☐ No ☐ Sometimes
S6	Does a hearing problem cause you difficulty when attending a party?	☐ Yes ☐ No ☐ Sometimes
E7	Does a hearing problem cause you to feel frustrated when talking to coworkers, clients, or customers?	☐ Yes ☐ No ☐ Sometimes
S8	Do a hearing problem cause you difficulties in the movies or theater?	☐ Yes ☐ No ☐ Sometimes
E9	Do you feel handicapped by a hearing problem?	☐ Yes ☐ No ☐ Sometimes

S10	Does a hearing problem cause you difficulty when visiting friends, relatives, or neighbours?	☐ Yes ☐ No ☐ Sometimes
S11	Does a hearing problem cause you difficulty hearing/understanding coworkers, clients, or customers?	☐ Yes ☐ No ☐ Sometimes
E12	Does a hearing problem cause you to be nervous?	☐ Yes ☐ No ☐ Sometimes
S13	Does a hearing problem cause you to visit friends, relatives, or neighbours less often than you would like?	☐ Yes ☐ No ☐ Sometimes
E14	Does a hearing problem cause you to have arguments with family members?	☐ Yes ☐ No ☐ Sometimes
S15	Does a hearing problem cause you difficulty when listening to TV or radio?	☐ Yes ☐ No ☐ Sometimes
S16	Does a hearing problem cause you to go shopping less often than you would like?	☐ Yes ☐ No ☐ Sometimes
E17	Does any problem or difficulty with your hearing upset you at all?	☐ Yes ☐ No ☐ Sometimes
E18	Does a hearing problem cause you to want to be by yourself?	☐ Yes ☐ No ☐ Sometimes
S19	Does a hearing problem cause you to talk to family members less often than you would like?	☐ Yes ☐ No ☐ Sometimes
E20	Do you feel that any difficulty with your hearing limits or hampers your personal or social life?	☐ Yes ☐ No ☐ Sometimes
S21	Does a hearing problem cause you difficulty when in a restaurant with relatives or friends?	☐ Yes ☐ No ☐ Sometimes

E22	Does a hearing problem cause you to feel depressed?	☐ Yes ☐ No ☐ Sometimes
S23	Does a hearing problem cause you to listen to TV or radio less often than you would like?	☐ Yes ☐ No ☐ Sometimes
E24	Does a hearing problem cause you to feel uncomfortable when talking to friends?	☐ Yes ☐ No ☐ Sometimes
E25	Does a hearing problem cause you to feel left out when you are with a group of people?	☐ Yes ☐ No ☐ Sometimes

Ventry, I. M., & Weinstein, B. E. (1982). The Hearing Handicap Inventory for the Elderly: A new tool. *Ear Hear, 3*, 128-134.

THE UNIVERSITY OF QUEENSLAND
AUSTRALIA

SOCIAL FUNCTION QUESTIONNAIRE

PLEASE LOOK AT THE STATEMENTS BELOW AND TICK THE REPLY THAT COMES CLOSEST TO HOW YOU HAVE BEEN RECENTLY

I complete my tasks at work and home satisfactorily.	☐ Most of the time<br>☐ Quite often<br>☐ Sometimes<br>☐ Not at all	0<br>1<br>2<br>3
I find my tasks at work and at home very stressful.	☐ Most of the time<br>☐ Quite often<br>☐ Sometimes<br>☐ Not at all	3<br>2<br>1<br>0
I have no money problems.	☐ No problems at all<br>☐ Slight worries only<br>☐ Definite problems<br>☐ Very severe problems	0<br>1<br>2<br>3
I have difficulties in getting and keeping close relationships.	☐ Severe difficulties<br>☐ Some problems<br>☐ Occasional problems<br>☐ No problems at all	3<br>2<br>1<br>0
I get on well with my family and other relatives.	☐ Yes, definitely<br>☐ Yes, usually<br>☐ No, some problems<br>☐ No, severe problems	0<br>1<br>2<br>3
I feel lonely and isolated from other people.	☐ Almost all the time<br>☐ Much of the time<br>☐ Not usually<br>☐ Not at all	3<br>2<br>1<br>0
I enjoy my spare time.	☐ Very much<br>☐ Sometimes<br>☐ Not often<br>☐ Not at all	0<br>1<br>2<br>3

Tyrer, P., Nur, U., Crawford, M., Karlsen, S., Mclean, C., Rao, B., & Johnson, T. (2005). The social functioning questionnaire: A rapid and robust measure of perceived functioning. *International Journal of Social Psychiatry*, *51*(3), 265-275.

THE UNIVERSITY OF QUEENSLAND
AUSTRALIA

MEDICAL OUTCOME SURVEY (MOS SF-36)

For each of the following questions, please circle the number that best describes your answer.

**1. In general, would you say your health is:**	
Excellent	1
Very good	2
Good	3
Fair	4
Poor	5
**2. Compared to one year ago,**	
Much better now than one year ago	1
Somewhat better now than one year ago	2
About the same	3
Somewhat worse now than one year ago	4
Much worse now than one year ago	5

3. The following items are about activities you might do during a typical day. **Does your health now limit you** in these activities? If so, how much? (Circle One Number on Each Line)

	**Yes, Limited a lot (1)**	**Yes, Limited a little (2)**	**No, Not limited at all (3)**
a. **Vigorous activities**, such as running, lifting heavy objects, participating in strenuous sports	1	2	3
b. **Moderate activities**, such as moving a table, pushing a vacuum cleaner, bowling, or playing golf	1	2	3
c. Lifting or carrying groceries	1	2	3
d. Climbing **several** flights of stairs	1	2	3
e. Climbing **one** flight of stairs	1	2	3
f. Bending, kneeling, or stooping	1	2	3

g. Walking **more than a mile**	1	2	3
h. Walking **several blocks**	1	2	3
i. Walking **one block**	1	2	3
j. Bathing or dressing yourself	1	2	3

4. During the **past 4 weeks**, have you had any of the following problems with your work or other regular daily activities **as a result of your physical health**?
(Circle One Number on Each Line)

	**Yes (1)**	**No (2)**
a. Cut down the amount of time you spent on work or other activities	1	2
b. **Accomplished less** than you would like	1	2
c. Were limited in the **kind** of work or other activities	1	2
d. Had **difficulty** performing the work or other activities (for example, it took extra effort)	1	2

5. During the **past 4 weeks,** have you had any of the following problems with your work or other regular daily activities **as a result of any emotional problems** (such as feeling depressed or anxious)? (Circle One Number on Each Line)

	**Yes**	**No**
a. Cut down the amount of time you spent on work or other activities	1	2
b. **Accomplished less** than you would like	1	2
c. Didn't do work or other activities as **carefully** as usual	1	2

**6. During the past 4 weeks, to what extent has your physical health or emotional problems interfered with your normal social activities with family, friends, neighbors, or groups?**	
Not at all	1
Slightly	2
Moderately	3
Quite a bit	4
Extremely	5

**7. How much bodily pain have you had during the past 4 weeks?**	
None	1
Very mild	2
Mild	3
Moderate	4
Severe	5
Very severe	6
**8. During the past 4 weeks, how much did pain interfere with your normal work (including both work outside the home and housework)?**	
Not at all	1
A little bit	2
Moderately	3
Quite a bit	4
Extremely	5

These questions are about how you feel and how things have been with you **during the past 4 weeks**. For each question, please give the one answer that comes closest to the way you have been feeling. (Circle One Number on Each Line)

9. How much of the time during the **past 4 weeks** . . .

	**All of the time**	**Most of the time**	**A good bit of the time**	**Some of the time**	**A little of the time**	**None of the time**
a. Did you feel full of pep?	1	2	3	4	5	6
b. Have you been a very nervous person?	1	2	3	4	5	6
c. Have you felt so down in the dumps that nothing could cheer you up?	1	2	3	4	5	6
d. Have you felt calm and peaceful?	1	2	3	4	5	6
e. Did you have a lot of energy?	1	2	3	4	5	6

	All of the time	Most of the time	A good bit of the time	Some of the time	A little of the time	None of the time
f. Have you felt downhearted and blue?	1	2	3	4	5	6
g. Did you feel worn out?	1	2	3	4	5	6
h. Have you been a happy person?	1	2	3	4	5	6
i. Did you feel tired?	1	2	3	4	5	6

**10. During the past 4 weeks, how much of the time has your physical health or emotional problems interfered with your social activities (like visiting with friends, relatives, etc.)? (Circle One Number)**	
All of the time	1
Most of the time	2
Some of the time	3
A little of the time	4
None of the time	5

11. How **TRUE or FALSE** is each of the following statements for you. (Circle One Number on Each Line)

	Definitely True	Mostly True	Don't Know	Mostly False	Definitely False
a. I seem to get sick a little easier than other people	1	2	3	4	5
b. I am as healthy as anybody I know	1	2	3	4	5
c. I expect my health to get worse	1	2	3	4	5
d. My health is excellent	1	2	3	4	5

Ware JE, Snow KK, Kosinski M, Gandek B. *SF-36® Health Survey Manual and Interpretation Guide*. Boston, MA: New England Medical Center, The Health Institute, 1993.

THE UNIVERSITY OF QUEENSLAND
AUSTRALIA

PET EXPECTATIONS INVENTORY

PLEASE ANSWER THE FOLLOWING QUESTIONS ABOUT THE ROLE YOU EXPECT THE HEARING DOG TO TAKE IN YOUR LIFE BY CIRCLING THE APPROPRIATE NUMBER (1-7) NEXT TO EACH QUESTION. PLEASE COMPLETE ALL ITEMS. DO NOT LEAVE QUESTIONS UNANSWERED.

		Strongly Agree						Strongly Disagree
A.	I expect the dog to be a companion for me.	7	6	5	4	3	2	1
B.	I expect the dog always to be there for me.	7	6	5	4	3	2	1
C.	I expect to talk to my dog.	7	6	5	4	3	2	1
D.	I expect my dog to make me feel better when I am sad or discouraged.	7	6	5	4	3	2	1
E.	I expect to stroke and cuddle my dog.	7	6	5	4	3	2	1
F.	I expect my dog to love me.	7	6	5	4	3	2	1
G.	I expect my dog to be a source of laughter.	7	6	5	4	3	2	1
H.	I expect my dog to be an interesting topic of conversation with friends and relatives.	7	6	5	4	3	2	1
I.	I expect to play with my dog.	7	6	5	4	3	2	1
J.	I expect my dog to protect me.	7	6	5	4	3	2	1
K.	I expect to teach my dog tricks.	7	6	5	4	3	2	1
L.	I expect to confide in my dog.	7	6	5	4	3	2	1
M.	I expect my dog to be a living thing for me to love.	7	6	5	4	3	2	1

Kidd, A.H., Kidd, R.M., & George, C.C. (1992). Veterinarians and successful pet adoptions. *Psychological Reports*, *71*, 551-557.
